# Protocols for isolating and characterizing polysaccharides from plant cell walls: a case study using rhamnogalacturonan-II

**DOI:** 10.1186/s13068-021-01992-0

**Published:** 2021-06-22

**Authors:** William J. Barnes, Sabina Koj, Ian M. Black, Stephanie A. Archer-Hartmann, Parastoo Azadi, Breeanna R. Urbanowicz, Maria J. Peña, Malcolm A. O’Neill

**Affiliations:** 1 Complex Carbohydrate Research Center, The University of Georgia, 315 Riverbend Road, Athens, GA 30602 USA; 2 The Department of Biochemistry and Molecular Biology, The University of Georgia, Athens, GA 30602 USA

**Keywords:** Rhamnogalacturonan-II (RG-II), Pectin, Plant cell wall, Borate diester, SEC-MALS, NMR spectroscopy

## Abstract

**Background:**

In plants, a large diversity of polysaccharides comprise the cell wall. Each major type of plant cell wall polysaccharide, including cellulose, hemicellulose, and pectin, has distinct structures and functions that contribute to wall mechanics and influence plant morphogenesis. In recent years, pectin valorization has attracted much attention due to its expanding roles in biomass deconstruction, food and material science, and environmental remediation. However, pectin utilization has been limited by our incomplete knowledge of its structure. Herein, we present a workflow of principles relevant for the characterization of polysaccharide primary structure using nature’s most complex polysaccharide, rhamnogalacturonan-II (RG-II), as a model.

**Results:**

We outline how to isolate RG-II from celery and duckweed cell walls and from red wine using chemical or enzymatic treatments coupled with size-exclusion chromatography. From there, we applied mass spectrometry (MS)-based techniques to determine the glycosyl residue and linkage compositions of the intact RG-II and derived oligosaccharides including special considerations for labile monosaccharides. In doing so, we demonstrated that in the duckweed *Wolffiella repanda* the arabinopyranosyl (Ara*p*) residue of side chain B is substituted at *O-*2 with rhamnose. We used electrospray-MS techniques to identify non-glycosyl modifications including methyl-ethers, methyl-esters, and acetyl-esters on RG-II-derived oligosaccharides. We then showed the utility of proton nuclear magnetic resonance spectroscopy (^1^H-NMR) to investigate the structure of intact RG-II and to complement the RG-II dimerization studies performed using size-exclusion chromatography.

**Conclusions:**

The complexity of pectic polysaccharide structures has hampered efforts aimed at their valorization. In this work, we used RG-II as a model to demonstrate the steps necessary to isolate and characterize polysaccharides using chromatographic, MS, and NMR techniques. The principles can be applied to the characterization of other saccharide structures and will help inform researchers on how saccharide structure relates to functional properties in the future.

**Supplementary Information:**

The online version contains supplementary material available at 10.1186/s13068-021-01992-0.

## Background

Plant cell walls are the major component of plant biomass and represent a largely under-utilized renewable resource for the generation of materials, fuels, and high-value chemicals. These walls are largely composed of cellulose, hemicelluloses, and pectins, as well as wall-bound proteins and lignin. These components dynamically interact at the molecular level to control the structure and mechanical characteristics of cell walls, which in turn are intimately involved in determining plant morphology and function from the cellular to tissue scale. Moreover, cell wall composition is directly related to how plants develop, reproduce, and thus provide food and materials. To date, considerable effort has been dedicated to dissecting the functional contributions of individual wall components through genetic and biomechanical studies of plant cell and organ growth. For example, cellulose exists in a microfibril form composed of coalesced glucan chains that provide a rigid, ‘load-bearing’ scaffold with high tensile strength and the ability to constrain the growth of plant cells [1]. The fibrils are believed to non-covalently interact with hemicelluloses to control wall biomechanics and the organization of the cellulose during wall expansion [2–4]. Early models of the cell wall predicted that the cellulose–hemicellulose scaffold was embedded in a pectin matrix that itself had only a limited effect on wall properties. However, there is now an increasing awareness that pectins are a dynamic type of polysaccharide that associate with cellulose and hemicelluloses to determine wall charge, porosity, rigidity and hydration [5–8].

Pectins are of particular importance for cell expansion [9, 10], fruit ripening and plant organ abscission [11], pollen dehiscence [12, 13], seed hydration [14, 15], and cell–cell adhesion [16–20]. Pectic structures vary considerably depending on plant taxonomy, tissue, and developmental stage. Pectins and pectin oligosaccharides act as developmental cues and elicitors in pathogen defense. Traditionally, pectins are used commercially as thickeners, stabilizers, and gelling agents [21]. Pectins also serve as a prominent component of dietary fiber for humans. More recently, pectins have become a target of efforts to produce plants with increased yield and reduced biomass recalcitrance [9, 22–24]. Pectins have also been shown to have the potential for the creation of edible food-packaging [25], the treatment of gastrointestinal disorders [26], and the removal of metals and contaminants in wastewater [27]. Thus, pectins in general are a family of polysaccharides that are unique in form and function. Nevertheless, their valorization has been limited by the challenges involved in determining their primary structures and relating how these structures contribute to their functional properties.

**Fig. 1:**
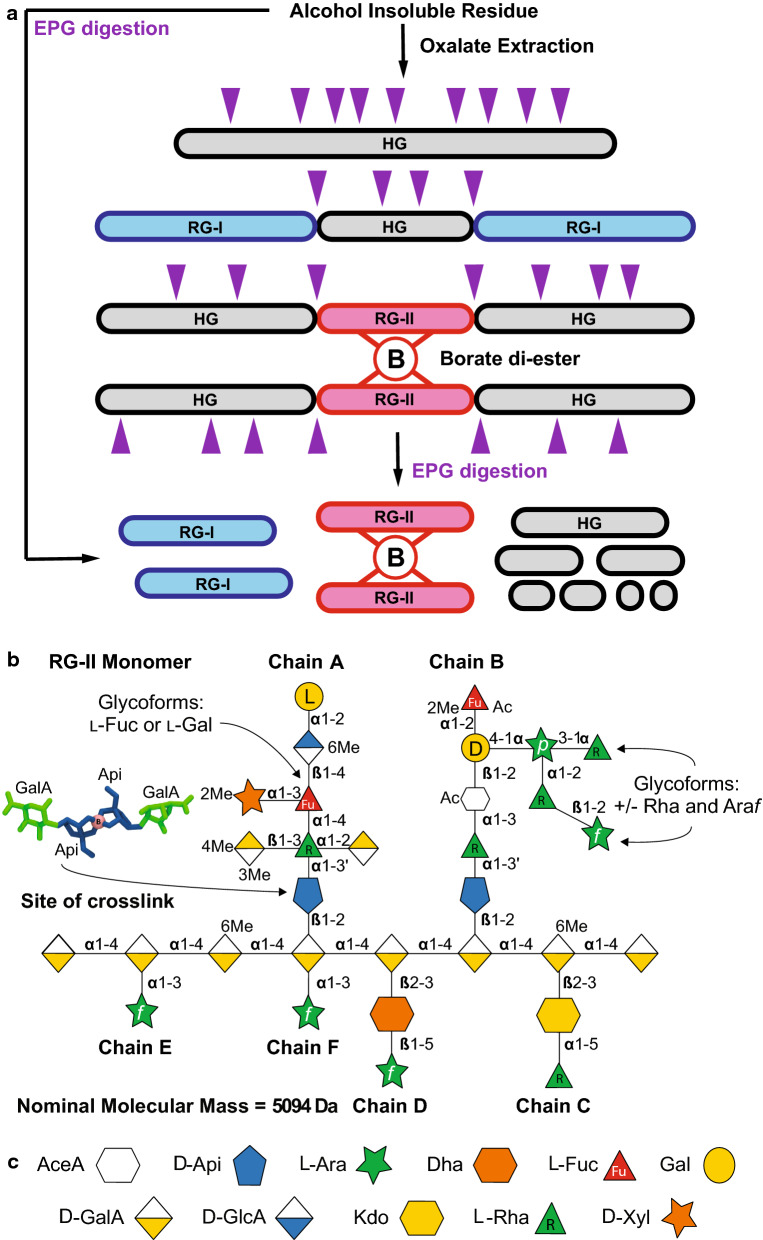
The isolation and glycosyl sequence of rhamnogalacturonan-II. **a** Schematic representation of the extraction of non-proteinaceous pectic domains including homogalacturonan (HG), rhamnogalacturonan-I (RG-I), and rhamnogalacturonan-II (RG-II) from alcohol insoluble residue (AIR). Covalently linked multi-domain pectin molecules can be released by oxalate extraction if necessary (duckweed preparation). AIR or isolated multi-domain pectin molecules are then treated with endopolygalacturonase (EPG) to hydrolyze the HG backbone to separate the pectic domains. Purple arrowheads represent sites of EPG cleavage on the HG backbone. **b** The glycosyl sequence of the rhamnogalacturonan-II (RG-II) monomer. The apiosyl residue involved in the formation of the borate diester cross-linked dimer is shown. Also shown are the sites of structural diversity in RG-II glycoforms isolated from different plants with or without Araf and Rha extensions of side chain B and l-Fuc instead of l-Gal in side chain A. The RG-II structure depicted, which includes all of the known non-carbohydrate substituents, has a nominal molecular mass of 5094 Da. **c** The RG-II-relevant symbols from the symbol nomenclature for glycans (www.ncbi.nlm.nih.gov/glycans/snfg.html) with minor modifications so they are accessible to people with color blindness

Pectins are classified into several general types—homogalacturonan (HG), rhamnogalacturonan-I (RG-I), rhamnogalacturonan-II (RG-II), apiogalacturonan (AGA), xylogalacturonan (XGA), and pectin-containing arabinogalactan protein (APAP1)—based on the structures of their backbone and substituents [28, 29]. HG, RG-II, AGA, and XGA have a backbone of 1,4-linked α-d-galacturonic acid (GalA) residues, which may be substituted with glycosyl and non-glycosyl substituents to form distinct pectin ‘domains’ (Fig. 1a) [29, 30]. For example, the structural diversity of HG results from the methyl-esterification of the carboxyl groups (*C-*6) of GalA and by *O*-acetylation of GalA at *O-*2 or *O-*3 [31]. In contrast, RG‐I has a backbone of the repeating disaccharide 4)-α-d-GalA-(1,2)-α-l-Rha-(1 that is substituted at *O-*4 of the rhamnose (Rha) by galactose (Gal) and arabinofuranose (Ara*f*)-containing side chains [29]. The backbone may also be *O*-acetylated.

RG-II (Fig. 1b) is the most structurally elaborate polysaccharide characterized to date and provides a compelling example of the byzantine nature of plant polysaccharides [32]. RG-II contains 11 different monosaccharides that are connected by at least 20 distinct glycosidic linkages (Fig. 1b, c). Further structural complexity is introduced by methyl-esterification, methyl-etherification, and *O*-acetylation of specific glycosyl residues [33]. Six side chains (termed A—F) branch off of the HG backbone, which has a degree of polymerization of at least eight GalA residues (Fig. 1b). Side chains A and B are oligosaccharides whereas C and D are disaccharides. E and F comprise a single α-l-Ara*f* residue linked to *O-3* of a backbone GalA or a backbone GalA that is also substituted at *O-2* with side chain A [34]. The structures of side chains C and D are conserved, whereas side chains A and B exhibit some structural diversity throughout vascular plants. Side chains A and B are linked to *O-2* of a backbone GalA via a β-d-apiosyl (Api) residue, but only the Api of side chain A has been shown to participate in the formation of the borate diester cross-linked RG-II dimer (Fig. 1b) [35]. Numerous studies have indicated the importance of RG-II in plant fitness and development and also demonstrate the relevance of glycan structures and glycoform variants in biology [19, 36–41].

In this work, we use RG-II as a case study to present a basic workflow and a set of complementary techniques that are necessary for the characterization of complex carbohydrates. RG-II presents a considerable challenge to the analyst because of its diverse glycosyl composition, the diversity of its glycosidic linkages, and the subtle structural variations that occur in different plants (Fig. 1b). We describe methods to extract and isolate RG-II from different sources and further detail how to characterize complex carbohydrates using established liquid chromatography, mass spectrometry (MS), and nuclear magnetic resonance spectroscopy (NMR) techniques. We use our characterization methods to introduce some caveats specific to RG-II analysis that may inform studies of other glycobiology researchers. Such methodologies are broadly applicable for the structural characterization of diverse pectic and hemicellulosic polysaccharides typically encountered in biomass obtained from different plants.

## Results and discussion

### Release of RG-II from the plant cell wall

RG-II accounts for between 1 and 5% by mass of the polysaccharides present in the primary cell walls of vascular plants [32]. RG-II can be extracted from isolated plant cell walls, which are prepared from plant tissues as their alcohol insoluble residues (AIR), using two related methods (see Fig. 1a). In our first protocol, high molecular mass pectins are solubilized by treating the AIR with 50 mM ammonium oxalate (Fig. 1a, Oxalate Extraction) [42]. Ammonium oxalate chelates divalent cations and solubilizes pectins held in the wall by calcium cross-links. Other chelators, including ethylenediaminetetraacetic acid (EDTA) and 1,2-Cyclohexylenedinitrilotetraacetic acid (CDTA), have also been used to solubilize cell wall pectins but have the disadvantage that they are not readily removed by dialysis against water, which may complicate further analyses [43]. The oxalate-solubilized pectins are then fragmented with endopolygalacturonase (EPG), which generates a mixture of RG-I, RG-II, and OGAs (Fig. 1a, EPG digestion). Ammonium oxalate extraction typically improves yields for samples such as duckweed AIR, while for other sources of RG-II including celery and Arabidopsis AIR, the ammonium oxalate extraction can be omitted. In this case, we use our second protocol, where we treat the AIR directly with EPG [44], which solubilizes RG-II together with RG-I and OGAs by fragmenting the HG backbone of pectic molecules (Fig. 1a).

### Isolation of RG-II from red wine

Red wine, which contains ~ 100 mg/L of RG-II, is a convenient source for isolating RG-II in gram amounts as the yeast involved in fermentation is unable to break down RG-II [45]. Red wine is first concentrated six-fold on a rotary evaporator and then ethanol is added to a final concentration of 80% (v/v). The mixture is kept overnight at 4 °C and the precipitate that forms is collected by centrifugation. The precipitate is dissolved in water and extensively dialyzed (3500 Da molecular weight cutoff (MWCO)) against water, and freeze-dried. This precipitate contains intact RG-II dimer, as well as arabinogalactan (AGP)/RG-I, oligogalacturonides (OGAs), and yeast-derived mannan [46].

### Isolation of RG-II by size-exclusion chromatography

**Fig. 2:**
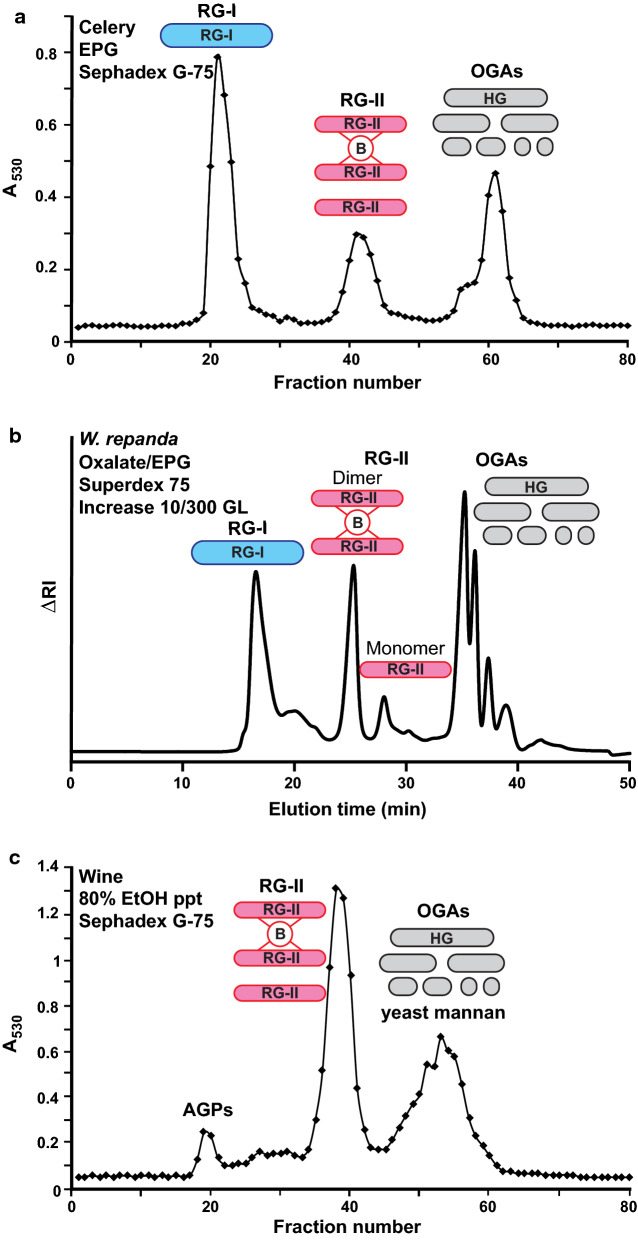
Isolation of RG-II using size-exclusion chromatography (SEC). **a** EPG-solubilized material (200–300 mg) from celery AIR in 50 mM NaOAc pH 5 was fractionated using a preparative Sephadex G-75 SEC column (1 m × 4 cm). Fractions were assayed colorimetrically for uronic acids (A_530_). **b** SEC of the EPG-treated oxalate-soluble fraction from *W. repanda* fractionated on a Superdex 75 Increase column monitored using refractive index (RI) detection. **c** The ethanol precipitated material from red wine (~ 500 mg) separated using a preparative Sephadex G-75 SEC column (1 m × 4 cm) and assayed colorimetrically for uronic acids (A_530_)

The EPG-solubilized material from cell walls and red wine precipitate contain a mixture of polysaccharides that can be separated by size-exclusion chromatography (SEC) to obtain material highly enriched in RG-II (Fig. 2). SEC of the EPG-solubilized wall extracts also gives fractions that contain RG-I and OGAs (Fig. 2a, b). In contrast, the wine precipitate (Fig. 2c) also gives fractions containing arabinogalactan proteins (AGPs), yeast mannan [46], and OGAs [45]. When large amounts of material (500–700 mg) are available, the precipitate from wine and the EPG-solubilized material from AIR can be fractionated by preparative scale SEC on a conventional Sephadex G-75 SEC column (4 × 100 cm) (Fig. 2a, c). In this case, large fractions (12 mL) are collected and analyzed colorimetrically for the presence of uronic acids [47]. If the amount of available pectic material is < 100 mg, as is the case for our small-scale preparations of duckweed RG-II, we use a Superdex 75 Increase GL10-300 column coupled with refractive index detection (Fig. 2b). We typically fractionate between 5 and 10 mg per run to maintain the separation efficiency of the column. RG-II monomer and dimer are readily resolved on the Superdex 75 column but not on the Sephadex G-75 column. Fractionation is complete in less than 1 h with the Superdex column and thus is much faster than the Sephadex G-75 column, which takes at least 12 h. However, approximately 50-fold more sample can be fractionated in a single run on the Sephadex G-75 column.

SEC separates compounds largely on the basis of their molecular mass and hydrodynamic volume, thus it is common for similarly sized molecules to co-elute with the target molecule during purification. It has been previously observed that the “crude RG-II” obtained from apple juice by ethanol precipitation was contaminated with galactan and arabinan [34], indicating that presence of diverse polysaccharides may be a common problem that affects the purity of RG-II preparations. Indeed, glycosyl residue composition analysis of the RG-II fraction isolated from celery AIR by preparative scale Sephadex G-75 SEC (Fig. 2a) showed that it contained far more galactose than expected based on the known structure of this pectic polysaccharide (Additional file 1: Table S1). We then found that the celery RG-II was contaminated with a galactan, which can be removed by anion-exchange chromatography (Additional file 1: Fig. S1 and Table S1). Thus, it is important to perform glycosyl residue composition analysis to determine the composition of glycans in fractions and to identify the presence of undesired components based on deviations from the expected ratios of monosaccharides for a given polysaccharide. Demonstrating purity and homogeneity can be challenging for other pectic polysaccharides, most notably RG-I, which often exists as a family of polysaccharides that differ in the abundance and types of side chains attached to the backbone [48]. In addition, linkage analysis can also provide information concerning the particular linkage of suspected contaminant monosaccharides which would indicate whether the particular monosaccharide is derived from RG-II or a different polysaccharide. Anion-exchange and size-exclusion chromatography alone or combined with additional enzymatic treatments that degrade the undesired component [34] may be sufficient to obtain homogeneous RG-II when purifying RG-II from fruit juices or plant cell wall extracts. Similar approaches for RG-I may result in the enrichment of a particular familiar of polymers with similar glycosyl compositions.

### Determining the abundance of the RG-II dimer and monomer

Most RG-II exists as a dimer in the plant cell wall. This dimer consists of two RG-II molecules that are covalently linked together by a borate diester formed between the apiosyl substituent of side chain A of each monomer (Fig. 1b). RG-II monomers and dimers are readily separated using a Superdex 75 10/300 column [35, 49]. We have found that they also separate on the recently introduced Superdex 75 Increase HR10/300 column, although their retention times may increase slightly. The amount of RG-II required for detection is determined in large part by the sensitivity of the refractive index detector used. In our experience, 50 μg of RG-II is sufficient for these analyses. This is fourfold less than the amounts (200 μg RG-II) we previously used [35, 49], and has increased our ability to compare the effects of different conditions including pH, buffers, and cations on dimer formation in vitro [33]. The absolute and relative abundances of RG-II monomer and dimer are estimated from their peak areas with respect to the known injection amount.

### Determining the molecular mass of RG-II using SEC with multi-angle static light scattering

Determining the molecular mass of polysaccharides is challenging as they are typically polydisperse polymers with a broad distribution of molecular weights and have a tendency to form aggregates. RG-II isolated from cell wall residues is an exception to this limitation as it is monodisperse and there is no evidence that it forms aggregates. These characteristics make RG-II an ideal molecule for polysaccharide molecular weight determination. The technique we used for this study involves SEC coupled to a multi-angle laser light scattering (MALS) detector and a refractive index (RI) detector (SEC-MALS). SEC-MALS provides an absolute molecular weight without reference to standards. However, these analyses first require that the specific refractive index increment (*dn/dc*) value of the molecule under investigation is determined. The *dn*/*dc* value is dependent on the composition of the polysaccharide of interest, the eluents used for SEC, temperature, and the wavelength of the light source of the refractive index detector. Thus, the *dn*/*dc* value should be calculated for each SEC-MALS system and polysaccharide. However, if it is not possible to obtain a pure polysaccharide for these calculations, published *dn*/*dc* values may prove to be adequate [50]. Here, purified celery RG-II monomer was used to calculate the *dn/dc* value, which was determined to be 0.122 ± 0.003 mg/mL.

Our SEC-MALS data (Table 1) show that the molecular mass of the celery RG-II monomer (M_w_ 4606 Da) is less than that of the similarly prepared monomer from red wine (M_w_ 4971 Da). These values are close to the calculated nominal mass of 5094 Da for the full RG-II monomer structure shown in Fig. 1b, which is typical of wine RG-II. Celery RG-II lacks the Ara*f* and two Rha that are attached to the Ara*p* of wine RG-II on side chain B (Fig. 1b) [33]. Thus, our SEC-MALS results are in general agreement with the structural differences between the celery and wine RG-II glycoforms. The native RG-II dimer isolated from wine and celery gave a polydispersity index of ~ 1, a value characteristic of monodisperse polymers, which suggests a uniform structure for the RG-II dimer in plant cell walls. SEC-MALS analysis also provides useful information in the study of other pectic polysaccharides, including the polydispersity and M_w_ of pectin and pectin fragments generated by enzymatic or selective chemical treatments.

**Table 1:** The molecular masses of celery and wine RG-II obtained by SEC-MALS

Source	RG-II	Mw (g/mol)	Mn (g/mol)	Polydispersity (Mw/Mn)
Celery	Dimer	9404 ± 143	9343 ± 176	1.01 ± 0.00
	Monomer	4606 ± 246	4556 ± 241	1.02 ± 0.02
Wine	Dimer	10,470 ± 428	10,378 ± 296	1.01 ± 0.01
	Monomer	4971 ± 307	4881 ± 186	1.01 ± 0.03

Data are averages and SD of at least three runs

### Glycosyl residue composition of RG-II

Once an RG-II-enriched fraction has been obtained by SEC, its glycosyl residue composition must be determined to verify the presence of RG-II and if substantial amounts of undesired polysaccharides are present, as discussed above. There is no single protocol that can be used to simultaneously detect and quantify all of the glycoses that comprise RG-II due to the chemical diversity of its constituent monosaccharides (Fig. 1b) [44, 51, 52]. Gas–liquid chromatography (GLC) analysis of the alditol acetate derivatives [53] is suitable for identifying and quantifying neutral glycoses and aceric acid (3-*C*-carboxy-5-deoxy-L-xylose, AceA; Table 2; Additional file 1: Fig. S2a).

**Table 2:** Partial glycosyl residue compositions of purified wine and celery RG-II

Glycose	Wine RG-II (Sephadex G-75 fraction)^a^	Celery RG-II (Q-Sepharose 1.5 M imidazole fraction)^b^
	Mol%	
MeFuc	2.9 ± 0.1	3.9 ± 0.4
Rha	16.3 ± 0.7	14.9 ± 0.9
Fuc	1.8 ± 0.0	2.9 ± 0.1
MeXyl	2.9 ± 0.2	4.0 ± 0.4
Ara	17.6 ± 1.0	14.1 ± 1.5
Api	3.6 ± 0.3	3.9 ± 0.8
AceA	1.2 ± 0.1	1.3 ± 0.3
Gal	17.9 ± 1.2	10.7 ± 1.0
Glc	1.4 ± 0.1	0.2 ± 0.0
GalA	29.2 ± 1.2	39.9 ± 3.8
GlcA	5.2 ± 2.6	4.1 ± 1.1

Quantification of neutral sugars and AceA was performed using alditol acetate derivatives. Quantification of uronic acids was performed using HPAEC-PAD. All values are expressed as a molar percentage and represent the average data from three replicates. See Additional file 1: Figure S2 for representative spectra

^a^The RG-II fraction isolated by SEC of the material solubilized by EPG treatment of celery petiole AIR (see Fig. 2 in the main text)

^b^The celery RG-II obtained by SEC was purified by anion-exchange chromatography. A solution of the celery RG-II (400 mg) in 10 mM imidazole–HCl pH 7 was applied to a column (15 cm × 2 cm; 47.1 mL column volume) of fast flow DEAE-Sepharose (Cytiva, USA). The column was eluted stepwise with 10 mM imidazole–HCl pH 7 (3 column volumes), 100 mM imidazole–HCl pH 7 (3 column volumes), and then with 1.5 M imidazole–HCl, pH 7 (4 column volumes). RG-II (~ 95% dimer, 295 mg) eluted with 1.5 M imidazole–HCl. A galactose-rich material (73 mg) eluted with 10 mM imidazole–HCl

**Fig. 3:**
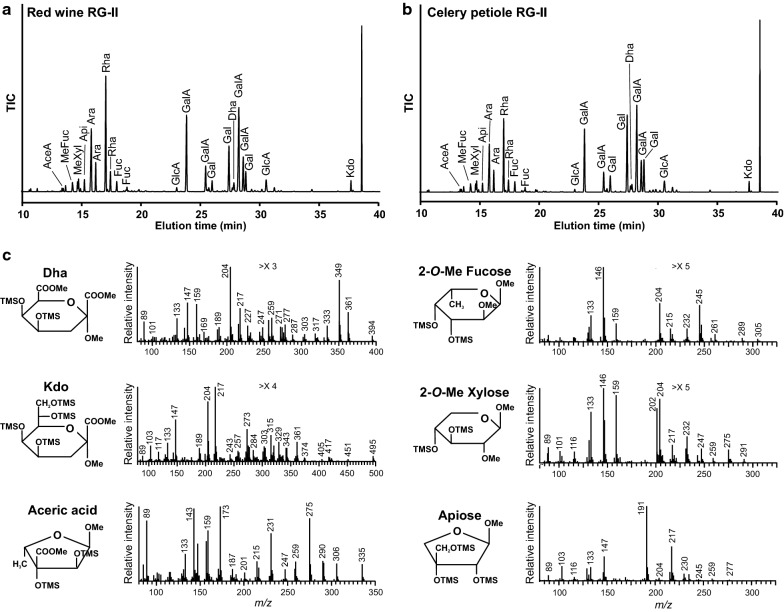
GLC-EI-MS total ion current profiles and selected mass spectra of the trimethylsilyl methyl-ester methyl glycoside derivatives of the monosaccharides generated from RG-II. **a**, **b** The GLC-EI-MS total ion current (TIC) profile of the TMS derivatives generated from wine (**a**) and celery (**b**) RG-II. The identity of the monosaccharide derivative in each peak is shown. The peak eluting at ~ 39 min is the TMS derivative of myo-inositol used as an internal standard. **c** The EI mass spectrum of selected monosaccharide derivatives generated from wine RG-II. The multiplication factor (> X) used to expand selected regions of each mass spectrum is shown

Two of the glycoses within RG-II, 2-keto-3-deoxy-*manno*-octulosonic acid (Kdo) and 3-deoxy-D-*lyxo*-2-heptulopyranosylaric acid (Dha), are degraded by the hot 2 M trifluoroacetic acid (TFA) treatment that is used to hydrolyze the glycosidic bonds and release the monosaccharides for the preparation of alditol acetate derivatives. Several modifications to this procedure are required to detect Kdo and Dha as their alditol acetates [51, 54]. Furthermore, it is difficult to quantify Dha due to a current lack of a commercially available standard. The alditol acetate procedure can also be modified to analyze hexuronic acids [55], but quantification can be challenging because of incomplete conversion of GalA to its corresponding lactone, which is subsequently reduced to galactitol and acetylated. Thus, GalA, glucuronic acid (GlcA), Kdo, and Dha are typically analyzed as their trimethylsilyl methyl-ester methyl glycoside (TMS) derivatives by GLC with electron impact mass spectrometry (GLC-EI-MS, see Fig. 3a, b) [56]. The Dha and Kdo derivatives have diagnostic mass spectra, which are used to confirm the identities of their peaks (Fig. 3c). The TMS derivatives of other glycoses characteristic of RG-II including AceA, 2-*O*-methyl-fucose (2MeFuc), 2-*O*-methyl xylose (MeXyl), and Api also give mass spectra that aid in their identification (Fig. 3c). Thus, these sugars can be used to identify RG-II containing peaks separated by SEC. Methyl-etherified GalA residues, which are present in RG-II from several plants, are challenging to quantify as they must be converted to their acetylated 6,6’-di-deuterio-galactitol derivatives prior to analysis by GLC-EI-MS [33]. Given the aforementioned complications, monosaccharide quantification may vary between samples but is nonetheless an indication of the composition of the saccharide of interest. Thus, a comprehensive description of all the RG-II monosaccharides requires GLC-EI-MS analyses of the alditol acetate (Table 2; Additional file 1: Fig. S2a) and TMS derivatives (Fig. 3) and can be further complemented by HPAEC-PAD analysis (Additional file 1: Fig. S2b) of the monosaccharides released by TFA hydrolysis if standards are available [42].

The glycosidic bonds of glycosyl residues in other pectins, notably RG-I, also exhibit differences in their susceptibility to hot acids. Thus, a compromise is often required between the amounts of a glycose released by hydrolysis and the amounts that are subsequently degraded by the hot acid [57]. Nevertheless, such differences in acid lability can be exploited in structural studies of RG-I. For example, selective acid hydrolysis of RG-I has been used to remove most of the Ara*f*-containing side chains and render the polysaccharide backbone susceptible to both rhamnogalacturonan hydrolase and lyase [58]. Similarly, selective removal of Api residues by autohydrolysis renders the backbone of apiogalacturonans susceptible to the action of endopolygalacturonase [59].

### Glycosyl-linkage analysis of the B side chain of RG-II

It is now accepted that even though the glycosyl sequence of RG-II is largely conserved, the structures of the A and B side chains are not identical in all plants (Fig. 1b) [33, 60–63]. Thus, a complete description of RG-II requires the characterization of these side chains. To this end, the A and B side chains are released from the RG-II backbone using selective hydrolysis with dilute aqueous TFA and characterized by mass spectrometry [60, 61, 64]. In our laboratory, we release side chain B by treating RG-II for 16 h at 40 °C with 0.1 M TFA. Small amounts of the A side chain may also be released with this treatment [60]. However, side chain A is released more effectively by treating RG-II for 1 h at 80 °C with 0.1 M TFA [60]. This treatment also releases side chain B but a substantial amount of the acid-labile terminal Ara*f* residue is hydrolyzed at the higher temperature.

**Fig. 4:**
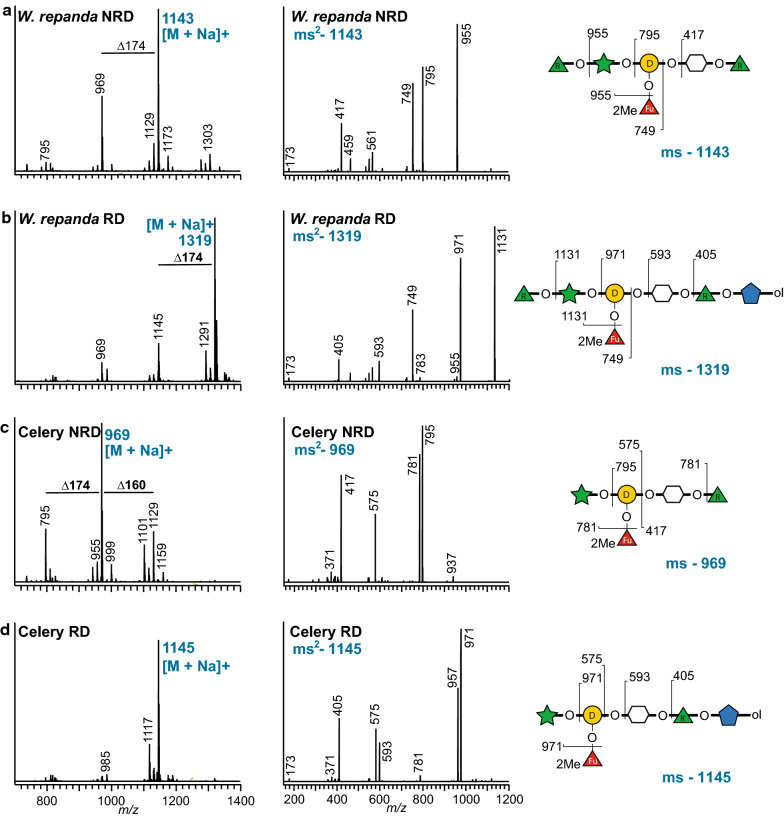
ESI–MS of side chain B released from RG-II by selective acid hydrolysis is degraded during methylation with solid NaOH and methyl iodide in DMSO. **a** Methylated reducing B side chain oligosaccharides generated from *W. repanda* RG-II (left panel). The ms^2^ spectrum (middle panel) and fragmentation pattern of the major ion (*m/z* 1143; right panel) is also shown. **b** NaBH_4_-reduced and methylated B side chain oligosaccharides generated from *W. repanda* RG-II (left panel). The ms^2^ spectrum (middle panel) and fragmentation pattern of the major ion (*m/z* 1319; right panel) is also shown. **c** Methylated reducing B side chain oligosaccharides generated from celery RG-II (left panel). The ms^2^ spectrum (middle panel) and fragmentation pattern of the major ion (*m/z* 969; right panel) is also shown. **d** NaBH_4_-reduced and methylated B side chain oligosaccharides generated from celery RG-II (left panel). The ms^2^ spectrum (middle panel) and fragmentation pattern of the major ion (*m/z* 1145; right panel) is also shown. Refer to Fig. 1c for the relevant symbol nomenclature for glycans

Side chain B glycoforms differ in the number and type of glycoses attached to the Ara*p* residue (Fig. 1b). For example, no sugars are attached to the Ara*p* in celery RG-II [12], whereas the Ara*p* is substituted at *O-2* with Rha or the disaccharide Ara*f*-Rha- and with Rha at *O-3* in the RG-II from many other plants (Fig. 1b) [61, 64, 65]. In a previous study, we reported that side chain B is a heptasaccharide in RG-II produced by several duckweeds, including *Wolffiella repanda* [42]. Matrix-assisted laser-desorption time-of-flight mass spectrometry (MALDI-TOF–MS) together with glycosyl residue composition analysis indicated that a single Rha residue is attached to the Ara*p* [42]; however, it was not determined if the Rha is attached to *O-*2 or *O-*3 of the Ara*p*. Electrospray (ESI) MS and MS^n^ in the positive ion mode is an established technique used to determine the mass of oligosaccharides, their glycosyl sequences, linkage positions, and the locations of *O*-acetyl and *O*-methyl substituents [66]. To this end, B side chain-enriched materials were generated from *W. repanda* and celery RG-II by treatment for 16 h with 0.1 M TFA at 40 °C and then isolated by SEC using a Superdex 75 column. The B side chain material was methylated using solid NaOH and methyl iodide in DMSO [67]. ESI–MS of the methylated products revealed that the predominant oligosaccharide from *W. repanda* (Fig. 4a) gave a [M + Na]^+^ ion at *m/z* 1143, which is consistent with an oligosaccharide composed of two Rha, one 2MeFuc, one Ara, one Gal, and AceA. The corresponding celery oligosaccharide gave a [M + Na]^+^ ion at *m/z* 969 (Fig. 4c), which is consistent with an oligosaccharide composed of one Rha, one 2MeFuc, one Ara, one Gal, and AceA. These masses are 160 atomic mass units lower than expected and correspond to the loss of a methylated pentose. We suspected that this was caused by the base-catalyzed degradation of the reducing Api during the methylation reaction. To confirm this, the *W. repanda* and celery side chain B-enriched fractions were treated with NaBH_4_ to convert the 3’-linked Api to 3’-linked apiitol. An apiitol at the former reducing end should not be susceptible to base degradation. The oligosaccharide-alditols were then per-*O*-methylated. The ESI–MS of the methylated oligosaccharide-alditol from *W. repanda* (*m/z* 1319, Fig. 4b) is consistent with a B side chain heptasaccharide composed of two Rha, one 2MeFuc, one Ara, one Gal, one AceA, and apiitol. The celery B side chain derivative (*m/z* 1145, Fig. 4d) is composed of one Rha, one 2MeFuc, one Ara, one Gal, one AceA, and apiitol. Thus, it is likely that the reducing form of side chain B undergoes base-catalyzed degradation during methylation with solid NaOH in DMSO. As such, we recommend that glycosyl-linkage analyses of oligosaccharides, irrespective of the polysaccharide they are generated from, are performed with the oligosaccharide-alditol rather than their reduced counterparts.

The ms^2^ spectra obtained by fragmenting the sodiated adducts of the methylated B side chains generated from *W. repanda* and celery RG-II (Fig. 4) are consistent with the known glycosyl sequences of side chain B from other plants (see Fig. 1b). However, the spectra contained few if any fragment ions that could be used to unambiguously determine the substitution pattern of the Ara*p*. Thus, we hydrolyzed the methylated material with 2 M TFA and converted the released methylated glycoses to their corresponding methylated alditols by reduction with NaBD_4_ [53]. These alditols were acetylated to give the per-*O*-methylated alditol acetate derivatives and analyzed by GLC-EI-MS (Additional file 1: Fig. S3). The identification of 1,2,5-tri-*O*-acetyl-3,4-di-*O*-methylarabinitol and 1,5-di-*O*-acetyl-2,3,4-tri-*O*-methylrhamnitol established that in *W. repanda* RG-II the Ara*p* is substituted at *O-2* with Rha.

### The non-glycosyl modifications of side chains A and B of RG-II

**Fig. 5:**
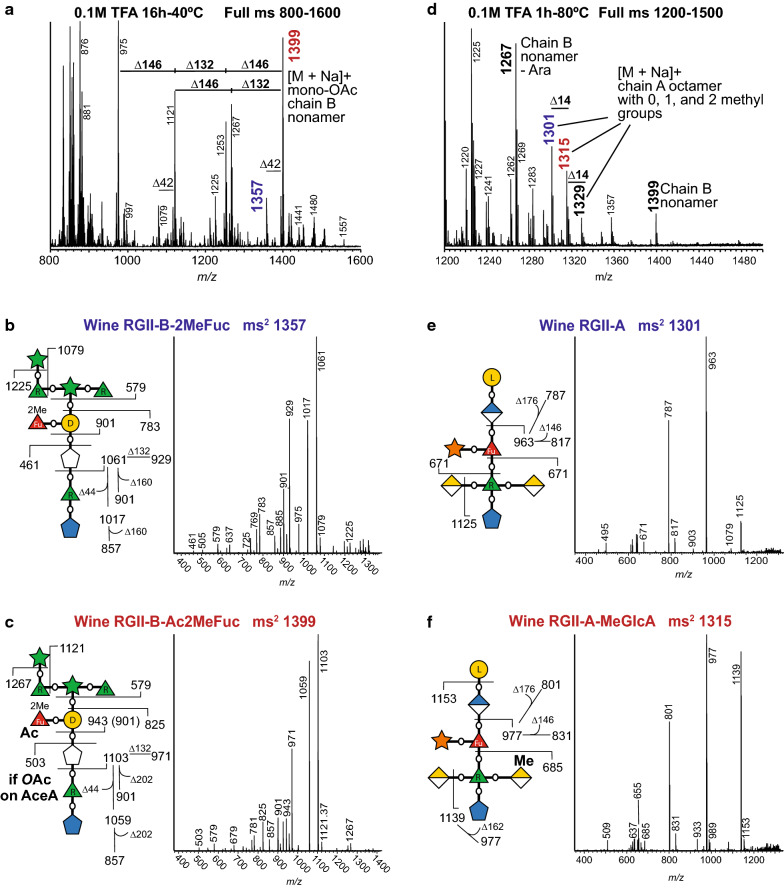
Locating the *O*-acetyl groups of side chain B and the methyl-ethers of side chain A using ESI–MS. **a** The ESI mass spectrum of the oligosaccharides generated by treating wine RG-II with 0.1 M TFA for 16 h at 40 °C (side chain B-enriched). ∆146 corresponds to a Rha residue, ∆132 corresponds to an Ara residue, and ∆42 corresponds to an *O*Ac group. **b** The ms^2^ spectra of the B side chain nonasaccharide (*m/z* 1357) and its fragmentation pattern. **c** The ms^2^ spectra of the B side chain mono-O-acetylated nonasaccharide (*m/z* 1399) and its fragmentation pattern. **d** The ESI mass spectrum of the oligosaccharides generated by treating wine RG-II with 0.1 M TFA for 1 h at 80 °C (side chain A-enriched). ∆14 corresponds to a methyl group. **e** The ms^2^ spectra of the A side chain octasaccharide (*m/z* 1301) and its fragmentation pattern. **f** The ms^2^ spectra of the A side chain mono-methylated octasaccharide (*m/z* 1315) and its fragmentation pattern. Refer to Fig. 1c for the relevant symbol nomenclature for glycans

In addition to its utility for determining glycosyl composition and linkage, electrospray ionization MS and MS^n^ in the positive ion mode have become the method of choice to obtain additional information concerning the precise locations of *O*-acetyl and *O*-methyl substituents present on saccharides [60, 61]. ESI–MS analyses showed that the mono-*O*-acetylated nonasaccharide (*m/z* 1399) accounts for a large portion of the B side chain that is released by treatment of red wine RG-II with 0.1 M TFA for 16 h at 40 °C (Fig. 5a). Small amounts of the di-*O*-acetylated chain B (*m/z* 1441) are also discernible. The side chain B spectrum also contains ions corresponding to the loss of a pentose (*m/z* 132) and a 6-deoxyhexose (*m/z* 146) from the mono-*O*-acetylated nonasaccharide. It is not known if these smaller side chain B oligosaccharides occur naturally in wine RG-II or are generated during treatment with warm dilute TFA. Side chain B is also released by treating RG-II with 0.1 M TFA for 1 h at 80 °C, but a substantial amount of the acid-labile terminal Ara*f* residue is hydrolyzed at the higher temperature (*m/z* 1267 in Fig. 5d). Compared to the ms^2^ spectrum of the unacetylated side chain B nonasaccharide (Fig. 5b), the ms^2^ spectrum of the mono-*O*-acetylated side chain B nonasaccharide (*m/z* 1399; Fig. 5c) contains ions at *m/z* 461, 825, and 943, suggesting that an *O*-acetyl group is located on the MeFuc or the Gal residue. The ms^2^ spectrum of the acetylated side chain B contains ions at *m/z* 901 and 857 (Fig. 5c) that are formed by loss of 202 amu (methyl 6-deoxy hexose + 1 *O*-acetyl group) from the ion at *m/z* 1103. The ions at *m/z* 901 and 857 are also present in the ms^2^ spectrum of the unacetylated nonasaccharide (Fig. 5b) and correspond to the loss of 160 amu (methyl 6-deoxy hexose) from the ion at *m/z* 1103. Thus, it is likely that the 2MeFuc residue is mono-*O*-acetylated. The presence of a low abundance ion at *m/z* 503 also suggests that in the mono-*O*-acetylated side chain B nonasaccharide, a small portion of the AceA is substituted with a single O-acetyl group (Fig. 5c). Thus, the *O*-acetylated B side chain from wine contains an *O*-acetyl group on two different sugars.

The glycosyl sequence of side chain A is largely conserved. However, the number of methyl groups attached to this side chain often differs between plants [33, 60, 61]. For example, red wine RG-II side chain A contains either 0, 1, or 2 methyl groups (Fig. 5d). The ms^2^ spectrum of the mono-*O*-methylated A side chain contains ions at *m/z* 685 and 1139 (Fig. 5f). These data, when taken together with the ms^2^ spectrum of wine side chain A lacking methyl groups (Fig. 5e), are consistent with the presence of a mono-*O*-methylated GalA [33, 61].

Techniques based on selective acid hydrolysis can be applied to the analyses of glycosyl and non-glycosyl substituents in other pectic polysaccharides. Selective acid hydrolysis is also an established method for fragmenting pectins. Moreover, numerous glycanases, many of them produced commercially, including exo- and endo-arabinanases and galactanases, galacturonases, and rhamnogalacturonases, are available and are now being widely used as tools to elucidate the structural features of pectins from diverse plants.

### NMR spectroscopy provides insight into RG-II methylation and acetylation

We described how to use MS techniques to characterize the A and B side chains of RG-II, but the complexity and chemical diversity of RG-II is not limited to these two side chains. Furthermore, MS-based techniques for characterizing oligosaccharides have the disadvantage of being destructive and typically require a prior enzymatic or chemical treatment to generate molecules suitable for analyses.

Plant polysaccharides are often further substituted by the addition of base-labile *O*-acetyl and methyl-esters and base-stable methyl-ethers, which adds to the complexity of their structures and may affect their functionality [21, 68]. RG-II contains mono-*O*-acetylated MeFuc and AceA [69] and often contains methyl-esterified GlcA on side chain A and methyl-esterified GalA in its backbone [33, 60, 61]. RG-II also contains three methyl-etherified glycoses: MeFuc, MeXyl, and MeGalA [33, 61]. These non-carbohydrate modifications are an additional source of structural diversity between RG-II glycoforms isolated from different plant species. In contrast to MS-based techniques, nuclear magnetic resonance spectroscopy (NMR) is a non-destructive technique particularly useful for characterizing intact glycans and their non-carbohydrate substituents. Here, we describe how ^1^H-NMR can be used to identify RG-II glycoses and non-glycosyl modifications.

**Fig. 6.**
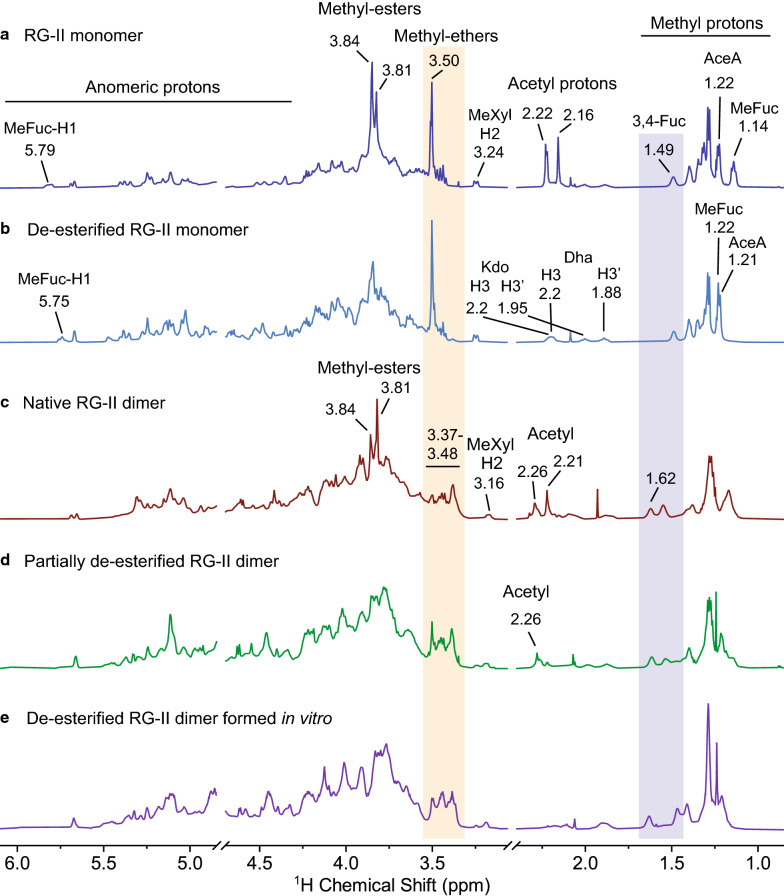
^1^H-NMR analysis of wine RG-II and the in vitro formed dimer. **a** Wine RG-II monomer. **b** De-esterified RG-II monomer. **c** Native RG-II dimer. **d** Partially de-esterified RG-II dimer. **e** RG-II dimer formed from the de-esterified RG-II monomer in vitro. The 1D ^1^H-NMR spectrum of the 1D ^1^H-NMR spectrum of the RG-II monomer (**a**) contains signals that correspond to acetyl and methyl groups. After base treatment, the signals of acetyl and methyl-esters are not observed in the spectrum of the de-esterified monomer (**b**), while the signals for methyl-ether remain. The removal of acetyl peaks revealed the presence of the diagnostic resonances of Kdo and Dha. The base treatment of the native dimer (**c**) removes methyl-esters but only partially de-acetylated the dimer (**d**). During in vitro dimerization of de-esterified monomer (**e**) several signals change. The more distinctive are the diagnostic resonances of methyl-ether (shaded orange) and 3,4 Fuc (shaded in blue), which have chemical shifts comparable to their counterparts in native and partially de-acetylated RG-II dimer

The ^1^H-NMR spectra of the native wine RG-II monomer and dimer (Fig. 6a, c) contain intense signals that correspond to methyl-ester protons (*δ* 3.81 and 3.84 ppm) and *O*-acetyl protons (*δ* 2.16 and 2.22 ppm). However, the presence of acetyl and methyl-esters complicate the ^1^H-NMR spectra of RG-II as they may cause changes in the chemical shifts of anomeric and ring protons of a monosaccharide. The methyl-ester and *O*-acetyl proton signals may also overlap with or obscure the signals of a glycosyl residue. Thus, to facilitate our NMR structural analysis, it was important to de-esterify RG-II. Since methyl and acetyl-esters are base labile, we routinely use cold 100 mM NaOH to de-esterify pectic polysaccharides, while minimizing the possibility of base-catalyzed degradation.

Somewhat unexpectedly, our ^1^H-NMR spectroscopic analyses showed that even though the base treatment fully hydrolyzed acetyl- and methyl-esters from the RG-II monomer (Fig. 6a, b), base treatment of the RG-II dimer hydrolyzed the methyl-esters but only resulted in partial de-*O*-acetylation of the dimer (Fig. 6c, d; Table 3). One signal corresponding to an *O*-acetyl group (δ 2.26 ppm) remained after base treatment of the RG-II dimer. Our MALDI-TOF–MS spectra of side chain B generated from the monomer, dimer, and their base-treated counterparts (Additional file 1: Fig. S4) provide additional evidence that side chain B is only partially de-*O*-acetylated by base treatment of the RG-II dimer. Together, these data show that in the dimer, the side chain B *O*-acetyl groups are not completely hydrolyzed by treatment with alkali.

**Table 3:** Selected signals in the 1H-NMR spectra of the RG-II monomer before and after base treatment

RG-II residue/group protons	^1^H chemical shifts (ppm)	
	RG-II monomer	Base-treated RG-II monomer
Methyl-ester protons	3.81, 3.84	nd
Acetyl protons	2.16, 2.22	nd
2Me-α-L-Fuc*p*-(1 → 2 H6	1.14	1.22
→ 2)-α-L-Ace*f*A-(1 → 3 H5	1.22	1.21
2Me-α-L-Fuc*p*-(1 → 2 H1	5.79	5.75
-5)-α-D-Kdo-(2 → 3 H3, H3’	1.88, nd	1.88, 2.2
**-**5)-β-D-Dha-(2 → 3 H3, H3’	1.95, nd	1.95, 2.2

*Nd* not determined

The removal of *O*-acetyl groups from the RG-II monomer revealed the presence of signals in the ^1^H-NMR spectrum that we assigned to the H3 and H3’ (*δ* 1.95 and 2.20 ppm) of Kdo and to the H3 and H3’ (*δ* 1.88 and 2.20 ppm) of Dha (Fig. 6b), which are challenging to identify by glycosyl residue composition analyses. These data also illustrate how the presence of acetyl- and methyl-esters complicate the NMR analysis of complex glycans. In addition, the ^1^H-NMR spectrum of the RG-II monomer both before (Fig. 6a) and after (Fig. 6b) de-esterification contained characteristic intensive signals that were assigned to base-stable methyl-ethers (*δ* 3.50 and 3.48 ppm) (Fig. 6a, b). It is notable that these signals are less intense in the native and base-treated dimer spectra (Fig. 6c, d). Together, these data demonstrate the utility of using NMR as a complementary approach to MS-based methods for the characterization of complex pectins.

### *Studying RG-II dimerization *in vitro* using SEC and NMR spectroscopy*

The methods discussed thus far provide the necessary tools to isolate RG-II and characterize its glycosyl and non-glycosyl components. When elucidating the role of RG-II in plants, we must consider not only its structure but the function of the molecule as it pertains to its cross-linking by borate (Fig. 1b). To study factors that affect RG-II dimerization, we first generate the RG-II monomer by treating the dimer (2.5 mg/mL) for 1 h at room temperature with 0.1 M HCl (Additional file 1: Fig. S5a). These conditions hydrolyze the borate diester but have no discernible effect on glycosidic linkages or non-glycosyl modifications. The acid is removed by dialysis against deionized water and the resulting monomer is freeze-dried. At this point, a stock solution of RG-II monomer in water (5 mg/mL) can be prepared and stored frozen. It is important to use known amounts of monomer since the rate and extent of dimer formation in vitro is sensitive to the concentrations of the monomer, boric acid, and divalent cation [33]. Our standard reaction mixture consists of RG-II monomer (50 μg) in 50 mM NaOAc pH 3.6 (50 μL) containing 1 mM boric acid and 0.5 mM Pb(NO_3_)_2_. The reaction is allowed to proceed at room temperature for at least 15 min and the products formed can be analyzed by SEC on a Superdex 75 column coupled with RI detection. We routinely use this procedure to determine the effects of pH, temperature, and cations on the rate of dimer formation [12]. For example, the presence of lead cations substantially increases the rate of dimer formation in vitro, whereas in the absence of lead the dimerization process may require at least 16 h to achieve significant conversion (Additional file 1: Fig. S5b, c) [33]. Pb(NO_3_)_2_ at concentrations above 1 mM is not soluble in some of the buffers used to investigate dimer formation, but can be replaced with an equivalent or higher concentration of SrCl_2_ or BaCl_2_. However, the Sr^2+^ and Ba^2+^ cations are not as effective at promoting dimerization as Pb^2+^ [49]. Somewhat surprisingly, we have found that adding Ca^2+^ does not increase the rate of dimer formation in vitro [35].

We also use ^1^H-NMR spectroscopy to study RG-II dimerization as it provides insight into the glycosyl residues that may have a role in the assembly process. For this procedure, wine RG-II dimer was converted to the monomer and then de-esterified. As mentioned above, de-esterification simplifies the NMR spectra, facilitating analysis of the dimerization process. The de-esterified monomer was then converted to the dimer by reaction with boric acid in the presence of Pb(NO_3_)_2_ in K phthalate, pH 4.0 (Fig. 6b, e). The most notable changes are in the signals corresponding to methyl-ethers present in the monomer (δ 3.5 ppm; Fig. 6b), which broadened after in vitro formation of the dimer and are comparable to their counterparts in native and partially de-acetylated RG-II dimer (Fig. 6c–e). Other signals related to methyl-ethers also change during dimerization. For example, the H-2 signal of 2-*O*-MeXyl shifted upfield from 3.24 to 3.16 ppm after in vitro dimerization, matching the chemical shift of the H-2 of 2-*O*-MeXyl observed in the native RG-II dimer (Fig. 6). These results demonstrate that the chemical environment of the methyl-ether attached to xylose changes substantially during dimer formation. However, the role of this substituent in dimer formation remains to be determined. Comparison of the dimer formed in vitro with its native counterpart (Fig. 6c–e), suggests that the RG-II formed *in planta* and in vitro have comparable structures. It is also notable that the dimer is readily formed from the de-*O*-acetylated monomer in vitro, which indicates that the *O*-acetyl groups are not required for the reaction to occur.

^1^H-NMR spectroscopy provides little information on the existence or nature of the borate cross-link since forming a borate-diol ester has only small effects on the ^1^H-NMR spectra of a glycose including Api [70–72]. However, boron NMR (^11^B-NMR) can be used to show that borate-diol esters are present in the RG-II dimer if ^11^B is present. Thus, combining the results of ^1^H- and ^11^B-NMR spectroscopy can provide abundant information on RG-II structure and chemical composition in a non-destructive manner.

## Conclusions

Plant cell walls contain polysaccharides with a rich chemical diversity that confer specific functions relevant to plant growth and development on the cellular and organ level. However, the complexity of polysaccharides has often hindered efforts to decipher their composition and functionality. In this work, we used RG-II as a model to outline the steps necessary to isolate and characterize complex polysaccharides. We demonstrate the utility of various types of chromatographies for isolating polysaccharides based on their physicochemical characteristics, and further show how MS- and NMR-based experiments are critical for determining complex glycan structures. The protocols we have described above can be applied to the characterization of most saccharide structures and will help inform researchers on how such structures relate to the functional properties of glycans.

## Materials and methods

### Chemicals and reagents

All chemicals and reagents were purchased from Sigma (Sigma, USA) unless otherwise noted. Dialysis was carried out using Spectrum™ Spectra/Por™, 3500 Dalton MWCO tubing (Spectrum Chemical Mfg Corp, USA).

### Plant material

*W. repanda* (ID9122) was obtained from the Rutgers Duckweed Stock Cooperative (Rutgers University, New Brunswick, NJ, USA). Plants were grown on 0.8% (w/v) agar pH 5.8, containing Schenk and Hildebrandt basal salts (1.6 g/L) and sucrose (1% w/v) in an Adaptis A1000 growth chamber (Conviron, Canada) at 19 °C and 15 °C with a 14 h light–10 h dark cycle, respectively, and a light intensity of 120 μmol quanta m^−2^ s^−1^. After 14 days of growth, the plants were washed from the surface of the agar using deionized water and kept at − 20 °C. Celery stalks (petioles) were purchased from a local supermarket.

### Preparation of cell walls

Cell walls were prepared from celery petioles and from *W. repanda* plants as their alcohol insoluble residues (AIR) as described [6]. Plant material can be harvested onto dry ice or into liquid nitrogen and kept at − 80 °C until required. For AIR preparation, plant material was suspended in aqueous 80% (v/v) ethanol and the tissue disrupted using a Polytron© homogenizer. The homogenate was then filtered through 100 μm nylon mesh and washed extensively with aqueous 80% ethanol. The homogenized tissue was suspended in chloroform:methanol (1:1 v/v) and stirred for a minimum of 1 h (or overnight) at room temperature and 125 rpm in a fume hood. The suspension was filtered through 100 μm nylon mesh, washed with chloroform:methanol, with acetone, and then air-dried. The final isolated residue is referred to as the alcohol insoluble residue (AIR). Starch was removed by treating suspensions of the AIR (in 0.5–1.0 g batches) in 50 mM sodium acetate (NaOAc) pH 5.2, for 24 h at 45 °C with the glucoamylase Spirizyme® (60 μL/g; Novozymes A/S, Denmark) and the α-amylase Liquozyme® (300 μL/g; Novozymes A/S, Denmark). The de-starched AIR was collected by filtration through nylon mesh (100 μm pore size, ThermoFisher, Waltham, MA, USA) and washed with deionized water.

### Isolation of pectins from plant cell walls and red wine

A pectin fraction containing RG-II was solubilized by treating *W. repanda* AIR (2 g) for 16 h at room temperature with 50 mM ammonium oxalate (250 mL) pH 6 [42]. The oxalate-soluble material was dialyzed (3500 Dalton MWCO) against deionized water and freeze-dried. A solution of the oxalate-soluble material (200 mg) in 50 mM NaOAc pH 5.2, was then treated for 24 h at 30 °C with endopolygalacturonase M2 (EPG) from *Aspergillus aculeatus* (2U Megazyme, Ireland). The solution was dialyzed (3500 Dalton MWCO) against deionized water and freeze-dried.

A suspension of celery AIR (25 g) in 50 mM NaOAc (1 L) pH 5.2, was treated for 24 h at 30 °C with EPG (5 U/g) with shaking at 150 rpm. The EPG-treated AIR was collected by filtration through nylon mesh (100 μm pore size) and retreated with EPG as before and filtered. The enzyme solubilized materials were combined, concentrated to ~ 100 mL by rotary evaporation at 37 °C, dialyzed (3500 Dalton MWCO) against deionized water, and freeze-dried.

Red wine (Yellow Tail Shiraz, 9 L) was concentrated to ~ 1.5 L by rotary evaporation at 37 °C. Absolute ethanol (3 L) was added and the mixture was kept overnight at 4 °C. The precipitate that formed was collected by centrifugation, dissolved in water, and re-precipitated by addition of ethanol to 75% (v/v). The second precipitate was dissolved in water, dialyzed (3500 Dalton MWCO), and freeze-dried (yield ~ 17.5 g).

### Purification of RG-II

Material enriched in RG-II was obtained by SEC of the EPG-treated oxalate-soluble material from *W. repanda* AIR*,* the EPG-soluble material from celery AIR, and the RG-II-containing precipitate obtained from red wine.

Briefly, solutions (500 μL) of the EPG-treated oxalate-soluble fraction (10 mg) from *W. repanda* in 50 mM ammonium formate pH 5 were filtered using nylon 0.45 μm Costar® Spin-X® centrifuge tube filters (Corning, USA). SEC was carried out using a Superdex 75 Increase column (Cytiva, Marlborough, MA, USA) at 0.5 mL/min with 50 mM ammonium formate pH 5, using a Dionex UltiMate 3000 pump (Thermo Scientific, USA). The column eluent was monitored with a Shodex RI-101 refractive index detector (Showa Denko America, USA). Fractions containing RG-II were collected manually and repeatedly freeze-dried to remove the ammonium formate.

A solution (10–12 mL) of the EPG-soluble material (200–300 mg) from celery in 50 mM NaOAc pH 5 was filtered as described above then fractionated by preparative SEC on a column (1 m × 4 cm) of Sephadex G-75 fine (Cytiva, USA) by elution with 50 mM NaOAc pH 5 at 1 mL/min. Fractions (12 mL) were collected. Aliquots (100 µL) of each fraction were assayed colorimetrically for uronic acids using the metabiphenyl assay [47] (see Fig. 2). Fractions enriched in RG-I, RG-II, and OGAs were separately pooled, dialyzed (3500 Dalton MWCO) against deionized water, and freeze-dried. Please see Additional file 1: Methods for protocols relevant to the purification of galactan from the RG-II-enriched SEC fraction from celery (Additional file 1: Materials and Methods).

The precipitate from red wine (~ 500 mg) was dissolved in 50 mM NaOAc pH 5 (10–12 mL), filtered through a 0.45 um nylon filter, and then fractionated using the preparative Sephadex G-75 SEC column by elution with 50 mM NaOAc pH 5 at 1 mL/min. Fractions (12 mL) were collected. Aliquots (100 µL) of each fraction were assayed colorimetrically for uronic acids using the metabiphenyl assay (Fig. 2) [47]. Fractions enriched in AGP/RG-I, RG-II, and OGAs were separately pooled, dialyzed (3500 Dalton MWCO) against deionized water, and freeze-dried.

### Preparation of RG-II monomer

The RG-II dimer was treated for 1 h at room temperature with 0.1 M HCl [49]. The solution was then dialyzed (3500 Dalton MWCO) against deionized water and freeze-dried. The RG-II dimer and monomer were chemically de-esterified by treatment for 16 h at 4 °C with 0.1 M NaOH. The solution was neutralized with acetic acid, dialyzed (3500 Dalton MWCO) against water, and freeze-dried.

The dimer was formed for NMR spectroscopic analysis by reacting the de-esterified wine monomer (2 mM) in 50 mM potassium hydrogen phthalate pH 4.0 (200 μl), for 16 h at 25 °C with 2 mM boric acid and 1.5 mM Pb(NO_3_)_2_.

### Determination of RG-II molecular mass using multi-angle light scattering coupled with size-exclusion chromatography (SEC-MALS)

SEC-MALS analysis was performed using an Agilent 1260 HPLC system (Agilent, USA) and a Superdex 75 10/300 SEC column (Cytiva, USA) connected in series to an Optilab T-rEX differential refractometer (Wyatt Technology Co., USA) operating at 25 °C and a Dawn Heleos 8 MALS detector (Wyatt Technology Co., USA) equipped with a He–Ne laser (λ = 660 nm). The column was eluted at a flow rate of 0.5 mL/min with 50 mM ammonium formate pH 5. All data were acquired and processed using ASTRA 7 software (Wyatt Technology Co., USA).

Native esterified wine and celery RG-II monomer and dimer (1–2 mg) were dissolved in ultrapure water and filtered using 0.45 μm nylon Costar® Spin-X® centrifuge tube filters (Corning, USA). The injection volume was 100 μL and a minimum of three injections for each polysaccharide were performed. The *dn*/*dc* value for RG-II was calculated by analysis of the purified native celery monomer. Different amounts of monomer (1.0, 0.5, 0.2, and 0.1 mg) were injected, and the *dn/dc* value was obtained on-line assuming 100% mass recovery for the peak of interest. The calculated *dn/dc* value (0.122 ± 0.003 mg/mL) was used for all RG-II analyses.

### Glycosyl residue composition analyses

Neutral glycosyl residue compositions were determined by GLC analysis of the alditol acetate derivatives after hydrolysis for 1.5 h at 120 °C with 2 M trifluoroacetic acid (TFA) [53]. Neutral monosaccharides and hexuronic acids were also analyzed by high-performance anion-exchange chromatography with pulsed amperometric detection (HPAEC-PAD) [42]. Briefly, RG-II (1–2 mg) was hydrolyzed for 1.5 h at 120 °C with 2 M TFA (250 μL). The hydrolysate was concentrated to dryness, dissolved in water (250 μL) and a portion (25 μL) analyzed HPAEC-PAD using a CarboPac PA1 column (Thermo Fisher, USA) using a Dionex ICS-3000 ion chromatography system (ThermoFisher, USA). The column was eluted at 1.0 mL min^−1^ with 32 mM NaOH (0–15 min) followed by a gradient of NaOAc (0–0.25 M) in 100 mM NaOH (15–35 min), a gradient of NaOAc (0.25–1 M) in 100 mM NaOH (35–45 min), and then with 1 M NaOAc in 100 mM NaOH (45–48 min). The column was equilibrated for 12 min with 32 mM NaOH prior to the next injection.

Neutral and acidic glycosyl residue compositions, including Kdo and Dha, were identified by analysis of the trimethylsilyl methyl glycoside derivatives as described [73]. Briefly, RG-II (250 μg) was suspended in methanolic 1 M HCl (250 μL) in screw-top glass tubes secured with Teflon-lined caps and heated for 18 h at 80 °C. After cooling to room temperature, the solutions were concentrated to dryness under a stream of nitrogen gas. The released methyl glycosides and methyl glycoside methyl-esters were then reacted for 30 min at 80 °C with Tri-Sil® (ThermoFisher, USA). GLC-EI-MS analysis of the TMS methyl glycosides was performed on an Agilent 7890A GC interfaced to an Agilent 5975C mass selective detector, with a Supelco Equity-1 fused silica capillary column (30 m × 0.25 mm ID).

### Generation of side chains A and B

Solutions of RG-II (~ 500 μg) in 0.1 M TFA (500 μL) were kept for 1 h at 80 °C and for 16 h at 40 °C. The solutions were concentrated to dryness under a flow of air and the residue washed with methanol (2 × 1 mL). The residues were dissolved in water (500 μL) and the acidic oligosaccharides isolated using graphitized carbon [74]. Briefly, Supelclean™ ENVI™-Carb cartridges (1 mL; Sigma, USA) were conditioned by washing with 2 ml of aqueous 80% (v/v) acetonitrile containing 0.1% TFA, followed by 5 ml of deionized water. The hydrolysates were then applied to the cartridge and the bound material washed with 2.5 mL of deionized water. Acidic oligosaccharides were then eluted with 3 mL of aqueous 50% acetonitrile containing 0.1% TFA, which was concentrated to dryness under a flow of warm air. The residue was dissolved in water (100–300 μL) and analyzed by ESI MS in the positive ion mode.

### Glycosyl-linkage analysis of W. repanda and celery side chain B

Fractions enriched in RG-II side chain B from *W. repanda* and celery RG-II were obtained by Superdex 75 SEC of the material generated by treatment of RG-II (~ 3 mg) with 0.1 M TFA (500 μL) for 16 h at 40 °C. One half of the fraction was then treated with NaBH_4_ (10 mg/mL 2 M ammonium hydroxide) to convert the oligosaccharides to their corresponding oligosaccharide-alditols. The oligosaccharides and the oligosaccharide-alditols were then separately per-*O*-methylated using solid NaOH in dimethylsulfoxide (DMSO) and methyl iodide [75]. Briefly, 200 μL of aqueous 50% NaOH was transferred to a screw-cap tube and then anhydrous methanol (200 μL) was added. The mixture was vortexed and then mixed with DMSO (4 mL). The suspension was centrifuged for 2 min at 2000×*g* and the DMSO and any white precipitate that formed was then carefully removed with a glass Pasteur pipette. This treatment was repeated a further four times until the pellet of solid NaOH was opalescent. The NaOH pellet was then suspended in DMSO (1.5 mL) and a portion (200 μL) was added to solutions of the oligosaccharide in DMSO (200 μL). The mixture was kept for 15 min at room temperature and then methyl iodide (100 μL) was added dropwise. After 30 min, water (1.5 mL) was added and the excess methyl iodide was then removed by gently bubbling nitrogen gas through the liquid. The methylated products were extracted into dichloromethane and portions analyzed by ESI–MS. The remaining material was converted to partially methylated alditol acetate derivatives and analyzed by GLC-EI-MS [53].

### Nanospray electrospray ionization mass spectrometry (ESI–MS)

Solutions of the native or per-*O*-methylated oligosaccharides (~ 1 mg/mL) were diluted tenfold with aqueous 50% methanol containing 0.1% v/v formic acid and directly infused at 0.5 μL/min into the nanospray ionization source of an Orbitrap Fusion Tribrid mass spectrometer (Thermo Fisher Scientific, USA) operated in the positive mode with an applied voltage of 1.9 kV. An automated program was used to collect a full mass spectrum and the MS^2^ of the highest intensity peaks. The top 300 peaks collected over an *m/z* range of 400–2000 were fragmented by collision-induced dissociation, with a dynamic exclusion of 60 s. The total run time was 20 min. Full mass spectra were collected at a resolution of 120,000 while the MS^2^ spectra were collected at a resolution of 60,000 [76]. Spectra were interpreted manually and with GlycoWorkbench 2 software.

### ^***1***^***H***-***NMR spectroscopy***

^1^H-NMR spectra were recorded with a Varian NMR spectrometer (Agilent Technologies) operating at 600 MHz using a 5 mm cold probe. RG-II samples were dissolved in D_2_O (0.2 mL, 99.9%; Cambridge Isotope Laboratories, Tewksbury, MA, USA) and placed in a 3 mm NMR tube. ^1^H-NMR spectra were obtained using standard Varian pulse programs. Chemical shifts were measured relative to internal DMSO (δH 2.721) or acetone (δH 2.225). Data were processed using MestReNova software (Mestrelab Research S.L., Spain).

## Supplementary Information

**Additional file 1.** Supplemental Materials and Methods, Supplemental Table S1, and Supplemental Figures S1 – S5.

## Data Availability

The datasets generated during the current study are available from the corresponding author on reasonable request.

## Abbreviations

AIR: Alcohol insoluble residue
Ara*f*: Arabinofuranose
Ara*p*: Arabinopyranose
AceA: Aceric acid, 3-*C*-carboxy-5-deoxy-L-xylose
AGA: Apiogalacturonan
AGP: Arabinogalactan protein
APAP1: Arabinoxylan pectin arabinogalactan protein1
Api: Apiose
^11^B-NMR: Boron nuclear magnetic resonance spectroscopy
CDTA: 1,2-Cyclohexylenedinitrilotetraacetic acid
Dha: 3-Deoxy-D-lyxo-2-heptulopyranosylaric acid
DHB: 2,5-Dihydroxybenzoic acid
DMSO: Dimethylsulfoxide
*dn/dc*: Specific refractive index increment
D_2_O: Dueterium oxide
EDTA: Ethylenediaminetetraacetic acid
EPG: Endopolygalacturonase
2MeFuc: 2-*O*-Me-l-Fucose
2MeXyl: 2-*O*-Methyl xylose
ESI–MS: Electrospray ionization mass spectrometry
Gal: Galactose
GalA: Galacturonic acid
GLC-EI-MS: Gas liquid chromatography with electron impact mass spectrometry
^1^H-NMR: Proton nuclear magnetic resonance spectroscopy
HPAEC-PAD: High-performance anion-exchange chromatography with pulsed amperometric detection
Kdo: 2-Keto-3-deoxy-manno-octulosonic acid
MALDI-TOF–MS: Matrix-assisted laser-desorption time-of-flight mass spectrometry
MALS: Multi-angle laser light scattering
MWCO: Molecular weight cutoff
MS: Mass spectrometry
NaBD_4_: Sodium borodueteride
NaOAc: Sodium acetate
NMR: Nuclear magnetic resonance spectroscopy
OGAs: Oligogalacturonides
RG-I: Rhamnogalacturonan I
RG-II: Rhamnogalacturonan-II
Rha: Rhamnose
RI: Refractive index
SEC: Size-exclusion chromatography
SEC-MALS: Size-exclusion chromatography with multi-angle laser light scattering
TFA: Trifluoroacetic acid
XGA: Xylogalacturonan
