# Supplementary material for: Protocols for isolating and characterizing polysaccharides from plant cell walls: a case study using rhamnogalacturonan-II
Source: Biotechnol Biofuels. 2021 Jun 22;14:142. doi: 10.1186/s13068-021-01992-0 (PMC8218411; doi:10.1186/s13068-021-01992-0)
Supplement: Supplementary file 1 — Additional file 1. Supplemental Materials and Methods, Supplemental Table S1, and Supplemental Figures S1 – S5. [file 13068_2021_1992_MOESM1_ESM.pdf]

Supplemental Materials and Methods

Purification of galactan from the RG-II-enriched fraction of EPG-solubilized material from celery AIR

The celery RG-II-enriched fraction obtained by SEC was further purified by anion-exchange chromatography. A solution of RG-II (400 mg) in 10 mM imidazole-HCl, pH 7 was applied to a column (15 cm x 2 cm; 47.1 ml column volume) of fast flow Q-Sepharose (Cytiva, USA). The column was eluted stepwise with 10 mM imidazole-HCl, pH 7 (3 column volumes), 100 mM imidazole-HCl, pH 7 (3 column volumes), and then with 1.5 M imidazole-HCl, pH 7 (4 column volumes). The total eluent for each concentration of imidazole-HCl was collected separately and then dialyzed (Spectrum™ Spectra/Por™, 3500 Dalton MWCO) against deionized water and freeze dried. RG-II (~95% dimer, 295 mg) was eluted with 1.5 M imidazole-HCl. A galactose-rich material (73 mg) eluted with 10 mM imidazole-HCl.

Characterization of the galactan that co-eluted with RG-II during SEC

Solutions of the galactan (5mg) in 50 mM NaOAc, pH 5.2 (0.5 mL) were treated for 16 h with a recombinant His-tagged *Geobacillus stearothermophilus*-1,4-β-D-galactanase [27] that was expressed in *Escherichia coli* and purified using a Ni-NTA column as described. The reaction mixture was then fractionated by SEC on a Superdex 75 column. The oligosaccharide products were collected and analyzed by MALDI-TOF MS.

Matrix assisted laser desorption ionization-time of flight mass spectrometry (MALDI-TOF MS)

MALDI-TOF mass spectra were obtained using a Microflex LRF mass spectrometer and workstation (Bruker, Billericay, MA, USA). Positive ion spectra were obtained using a stainless steel MALDI target plate with 2,5-dihydroxybenzoic acid (DHB, 1 µL, 10 mg/mL in aq. 50% (v/v) acetonitrile) as the matrix. The matrix (1 µL) and analyte (1 µL) were mixed directly on the plate and concentrated to dryness using a flow of warm air from a hair dryer. Spectra from at least 200 laser shots were summed to generate each mass spectrum.

Supplemental Table and Figures

Table S1. Glycosyl residue compositions of celery RG-II before and after the removal of galactan by anion-exchange chromatography.

Glycose	RG-II (G-75 fraction)[1]	RG-II (Q-Sepharose 1.5 M imidazole fraction)[2]	Galactan (Q-Sepharose 10 mM imidazole fraction)[2]
		Mol%	
MeFuc	4	8	nd
Rha	15	27	2
Fuc	3	6	nd
MeXyl	4	8	nd
Ara	17	19	6
Api	4	9	nd
AceA	2	4	nd
Gal	51	17	92
Glc	2	1	nd

[1]The RG-II fraction isolated by SEC of the material solubilized by EPG treatment of celery petiole AIR (see Fig. 2 in the main text).

[2]The celery RG-II obtained by SEC was purified by anion-exchange chromatography. A solution of RG-II (400 mg) in 10 mM imidazole-HCl pH 7 was applied to a column (15 cm x 2 cm; 47.1 mL column volume) of fast flow Q-Sepharose (Cytiva, USA). The column was eluted stepwise with 10 mM imidazole-HCl pH 7 (3 column volumes), 100 mM imidazole-HCl pH 7 (3 column volumes), and then with 1.5 M imidazole-HCl, pH 7 (4 column volumes). Each fraction was dialyzed (Spectrum™ Spectra/Por™ 3500 Dalton MWCO) against deionized water, freeze dried, and glycosyl composition was determined.

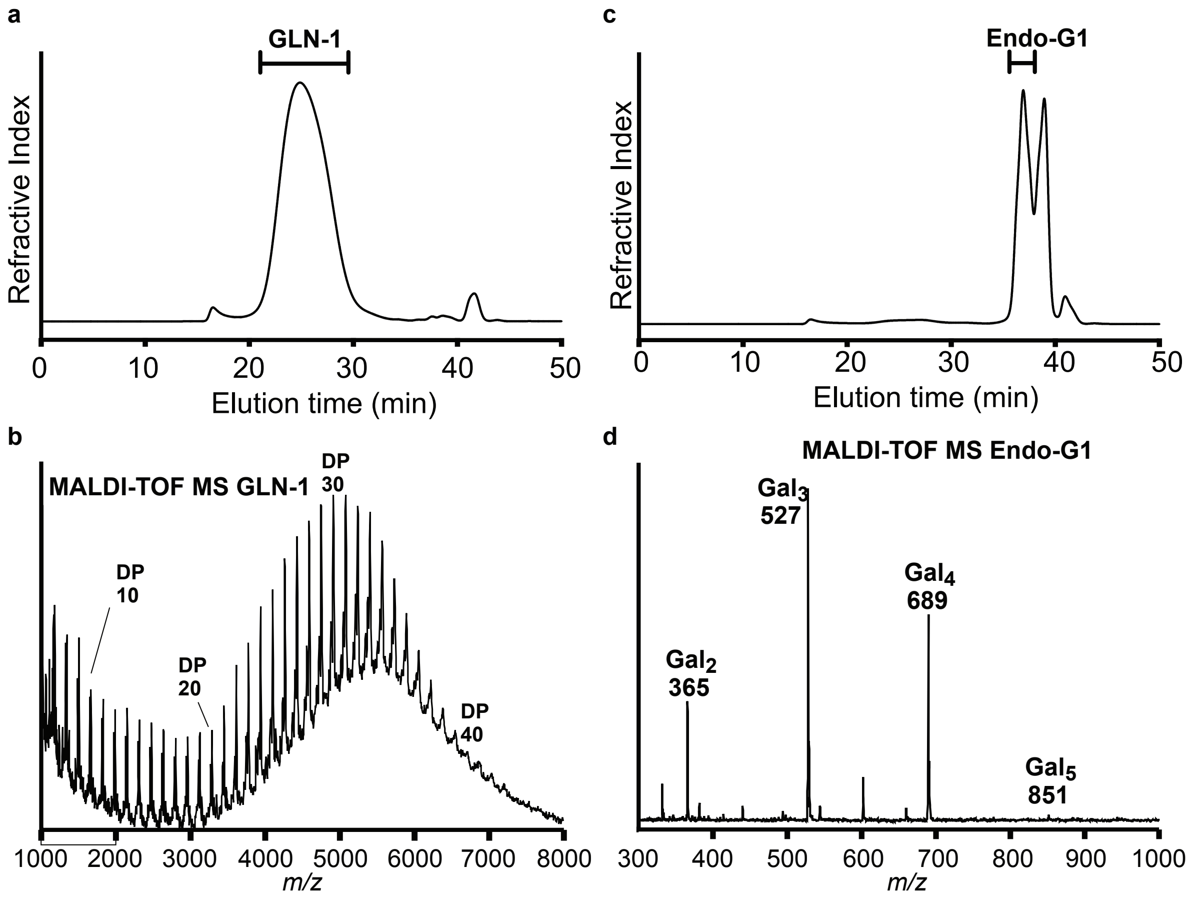

Figure S1. Characterization of the galactan-enriched material from celery EPG extracts that co-elute with RG-II. a. The galactose-rich fraction was separated from RG-II by anion-exchange chromatography and then fractionated on a Superdex-75 column as shown. b. MALDI-TOF MS analysis of Fraction GLN-1 indicates the fraction contains a broad distribution of hexose-derived oligosaccharides with DPs between 7 and 40. Glycosyl-linkage analysis of Fraction GLN-1 showed that at least 90% of the Gal is 4-linked. c. Fraction GLN-1 was treated with a recombinant endo-1,4-ß-galactanase and the products formed were fractionated by SEC on a Superdex-75 column. d. MALDI-TOF MS analysis of Fraction Endo-G1 shows the presence of oligosaccharides with DPs between 2 and 5. We conclude that the material that co-elutes with celery RG-II is predominantly a 1,4-linked ß-D-galactan.

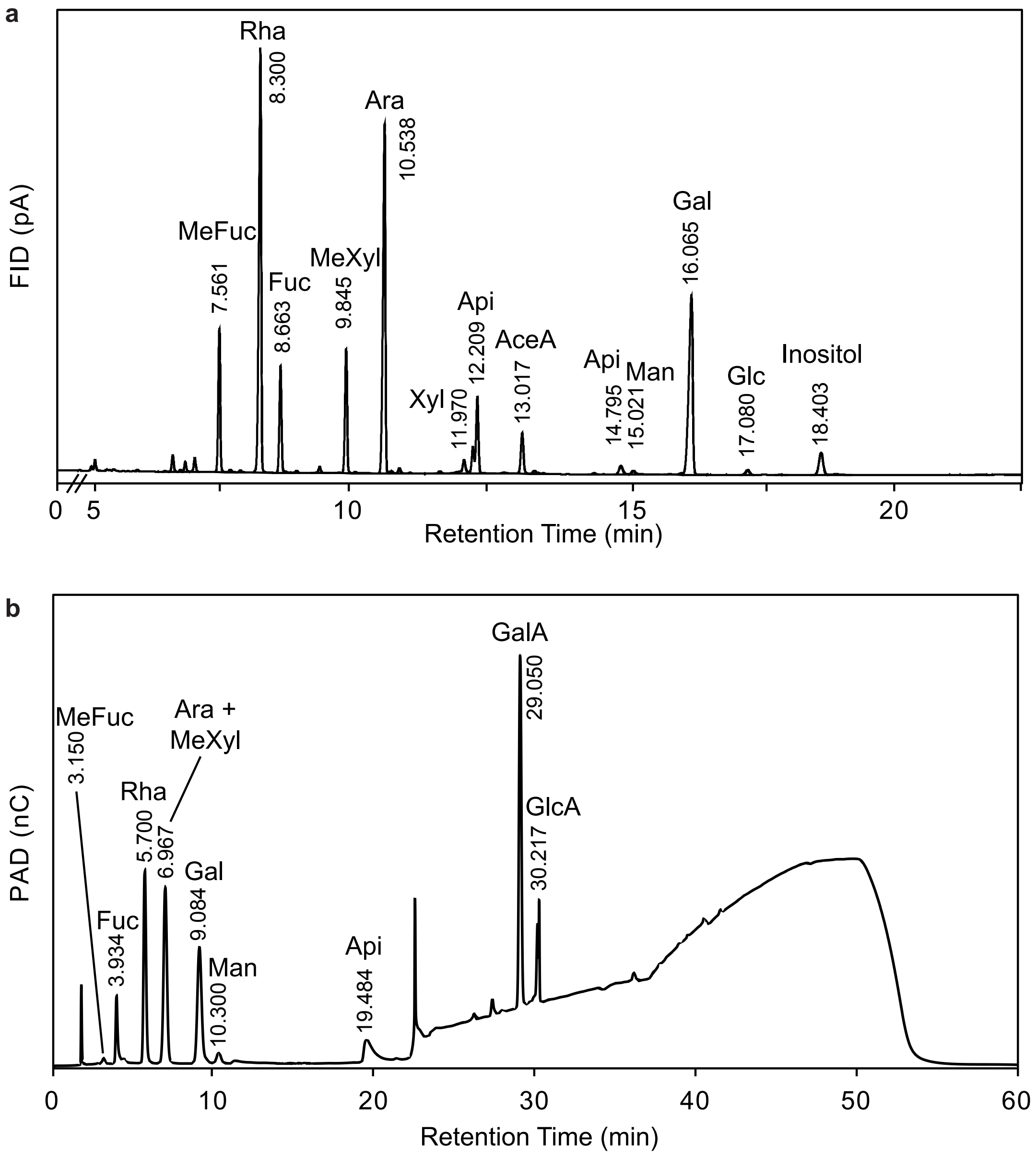

Figure S2. Monosaccharide composition analysis of hydrolyzed RG-II. a. GLC spectrum of RG-II-derived alditol acetates of neutral sugars, AceA, and an inositol standard. b. HPAEC-PAD spectrum of TFA-hydrolyzed RG-II-derived monosaccharides including uronic acids.

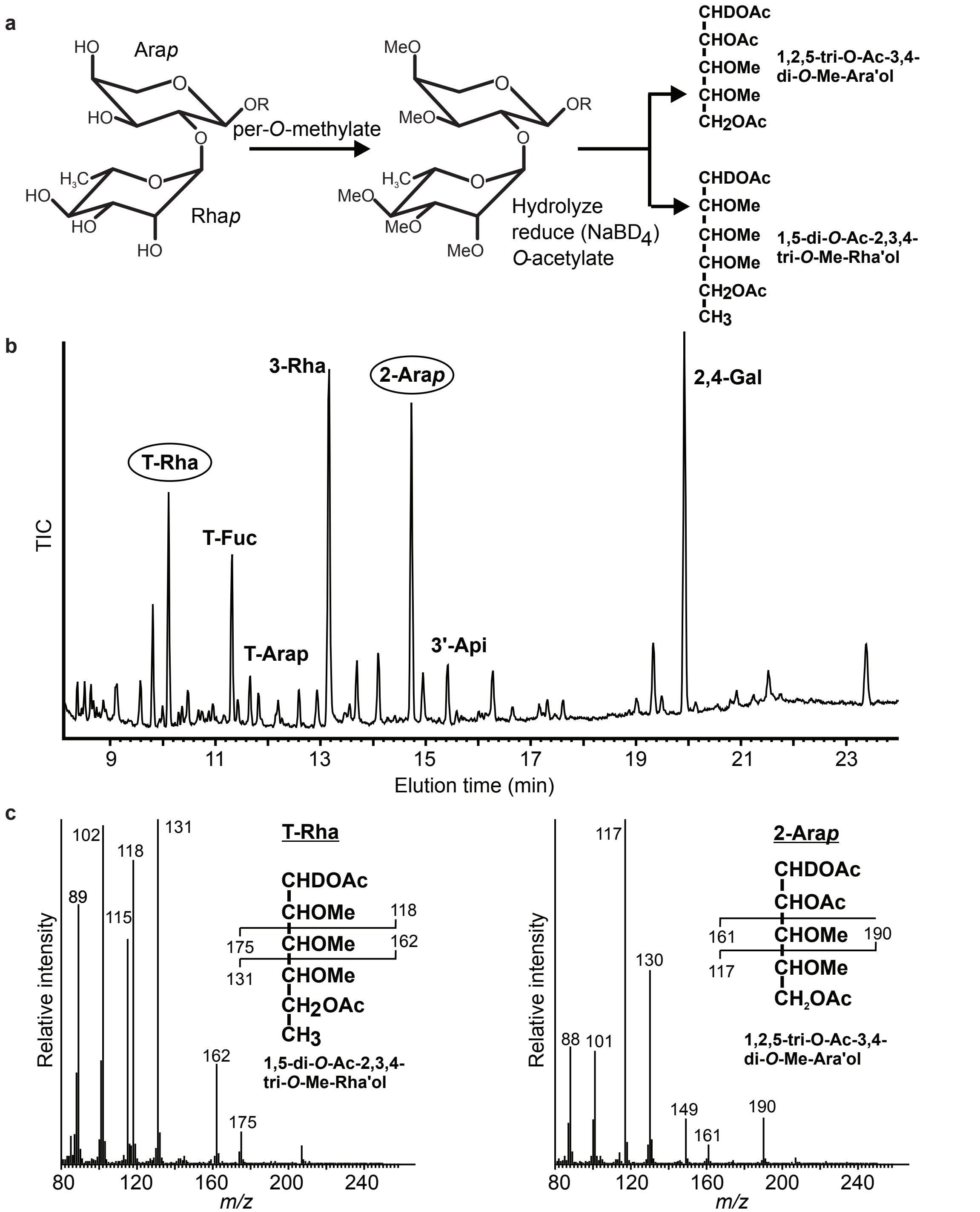

Figure S3. Determining the linkage of the Ara*p* residue in side chain B of *W. repanda* RG-II. a. Schematic showing the procedure used to generate the per-*O*-methylated alditol acetate (PMAA) derivatives used for GLC-EI-MS analysis. b. The GLC-EI-MS total ion current profile of the PMAAs generated from side chain B of *W. repanda* RG-II. T-Rha is a terminal non-reducing rhamnosyl. c. The EI mass spectra of 1,5-di-*O*-Ac-2,3,4-tri-*O*-Me-rhamnitol and 1,2,5-tri-*O*-Ac-3,4-di-*O*-Me-arabinitol. These data show that the Ara*p* is substituted at the *O*-2 with a terminal Rha*p*.

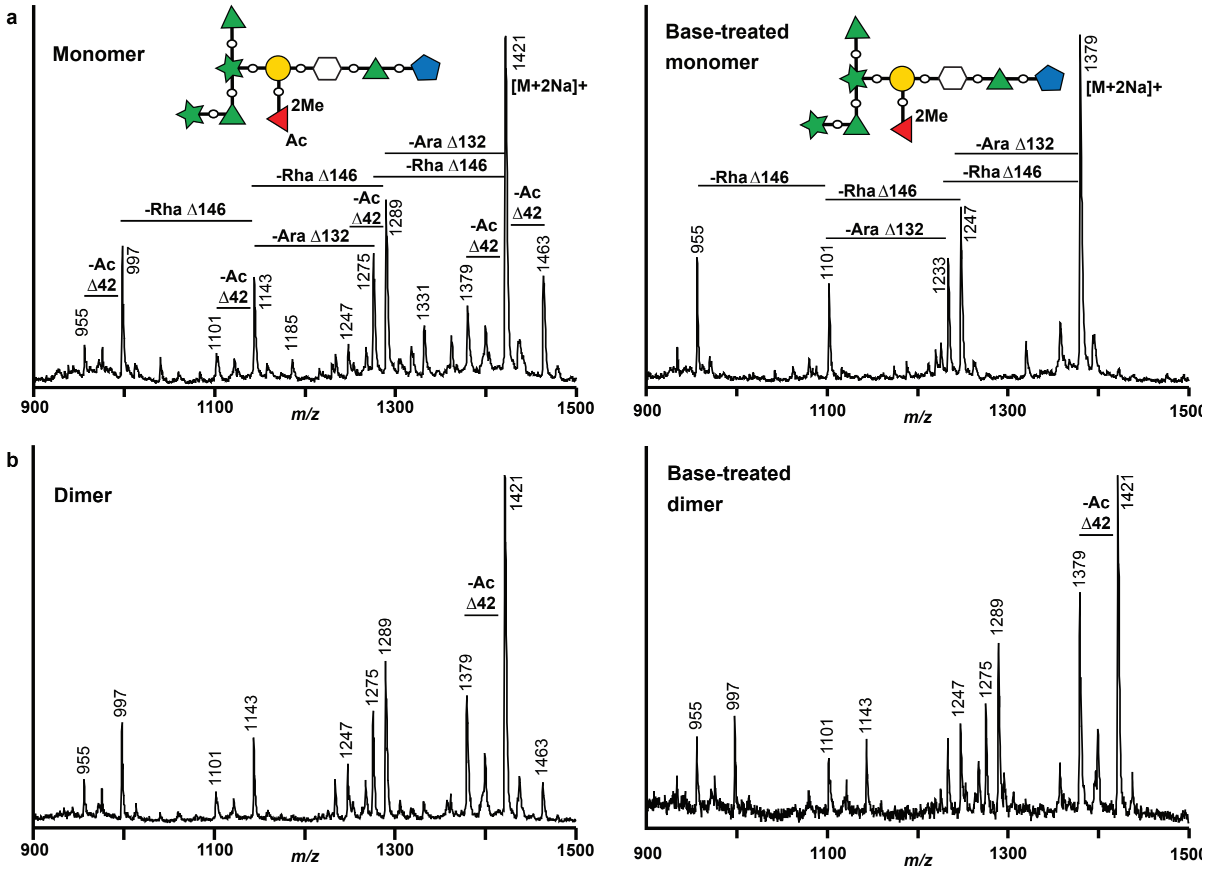

Figure S4. MALDI-TOF mass spectra of side chain B generated from native and de-esterified RG-II monomer and dimer. a. The top two panels show the MALDI-TOF mass spectra of side chain B from the native monomer and dimer. The ion at *m/z* 1421 corresponds to the $[M + 2Na]^+$ ion of the mono-*O*-acetylated nonasaccharide. b. The bottom two panels show the MALDI-TOF mass spectra of side chain B from the de-esterified monomer and dimer. The ion at m/z 1379 in the monomer spectrum corresponds to the [M + 2Na]+ ion of the nonasaccharide. A strong signal for the mono-*O*-acetylated nonasaccharide is clearly discernible in the mass spectrum of side chain B from the base-treated dimer. MALDI-TOF mass spectra were obtained in the positive ion mode. Spectra from at least 200 laser shots were summed to generate each mass spectrum. Refer to Fig. 1c for the relevant symbol nomenclature for glycans.

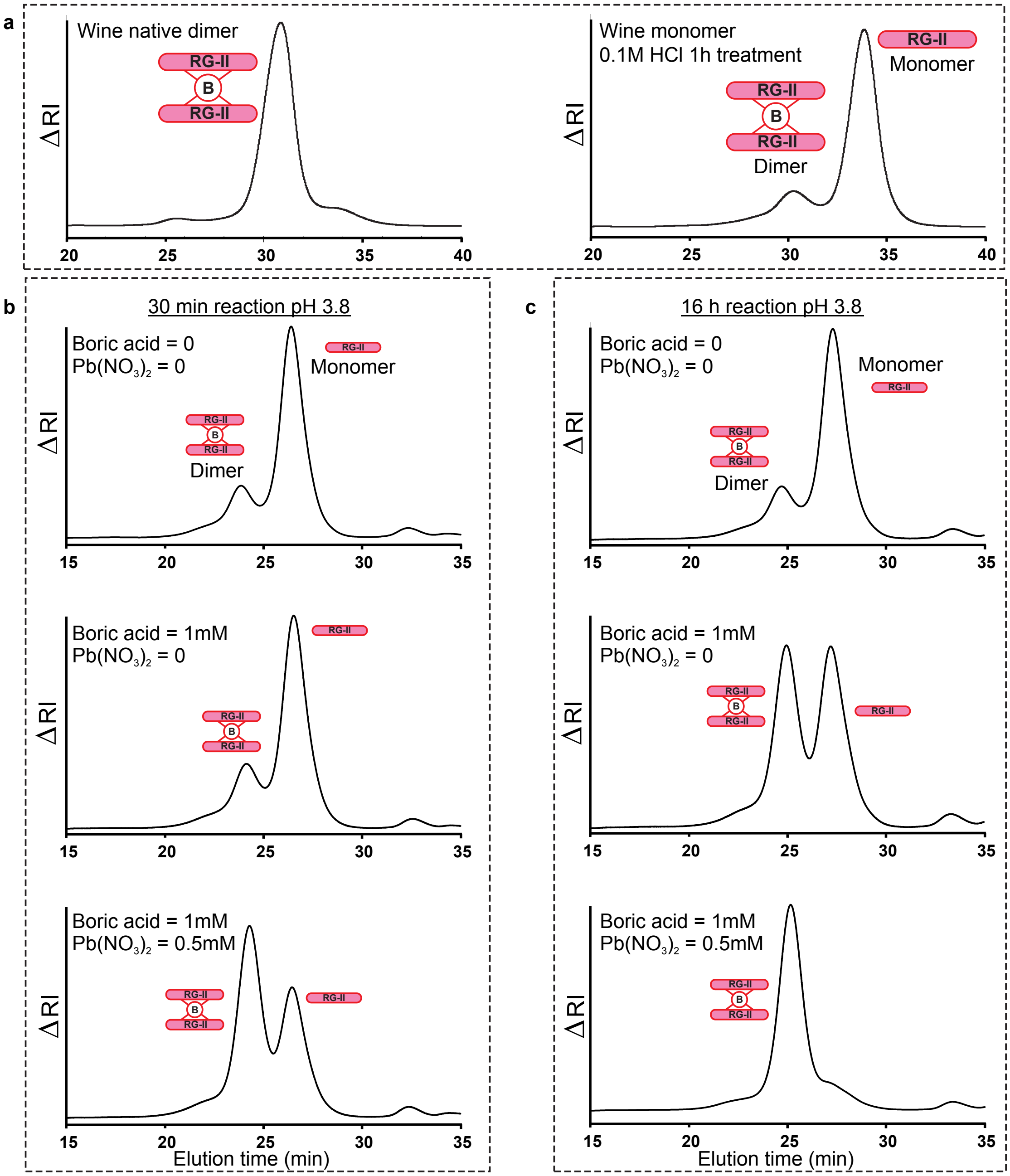

Figure S5. Interconversion of the RG-II dimer and monomer. a. The left panel shows the SEC elution profile of the dimer on a Superdex 75 Increase 10/300 column. The right panel shows the SEC elution profile of the monomer on a Superdex Increase 10/300 column. The monomer was formed by treating the dimer for 1 h with 0.1M HCl. b. The left top, middle, and bottom panels show the abundance of the dimer after reacting the monomer for 30 min at pH 3.8 with different concentrations of boric and $Pb(NO_3)_2$. Each reaction contained 0.2 mM RG-II monomer. c. Top right, middle, and bottom panels show the abundance of the dimer after reacting the monomer for 16 h at pH 3.8 with different concentrations of boric and $Pb(NO_3)_2$. Each reaction contained 0.2 mM RG-II monomer. The mixtures were separated on a Superdex 75 10/300 column, which has faster elution times than the Superdex 75 Increase column. Note that the retention times in panel a (recently introduced Superdex 75 Increase HR10/300 column) differ from those in b and c (older Superdex 75 HR10/300 column).
